# Results of Deep Surgical Site Infections Treated with the Debridement, Antibiotics, and Implant Retention (DAIR) Protocol: 25 Cases

**DOI:** 10.3390/jcm15124736

**Published:** 2026-06-18

**Authors:** Ali İhsan Ökten, Saygı Uygur, Emre Bilgin, Abdullah Kılıç, Kemal Şüheda Özkavaklı, Fatih Çiçek, Erencan Kılcı, Mehmet Babaoğlan, Şahin Sancaktar, Baran Uyanık, Ali Harmanoğullarından

**Affiliations:** 1 Department of Neurosurgery, University of Health Sciences, Adana City Training and Research Hospital, 01230 Adana, Turkey; dremreblgn@gmail.com (E.B.); klcabdullah@hotmail.com (A.K.); ksuhedaozkavakli@gmail.com (K.Ş.Ö.); fatih.cicek0147@gmail.com (F.Ç.); erencanadanademir@icloud.com (E.K.); drmehmetbabaoglan@gmail.com (M.B.); 0202sahin0202@gmail.com (Ş.S.); tripato02@outlook.com (B.U.); aliharmanoglu@gmail.com (A.H.); 2 Department of Neurosurgery, Kadirli State Hospital, 80750 Osmaniye, Turkey; uygursaygi@gmail.com

**Keywords:** DAIR protocol, spinal implant, deep surgical site infection, risk factors, spinal surgery, instrumentation

## Abstract

**Background/Objectives**: There is no consensus on whether it is possible to preserve implant retention during deep surgical site infections (SSIs), and there is no widely accepted treatment protocol to date for these patients. The aim of this study is to evaluate the efficacy of the debridement, antibiotics, and implant retention (DAIR) protocol in patients who were treated for degenerative thoracolumbar spinal disorder using spinal instrumentation. **Methods**: This retrospective study describes the 24-month outcomes of deep SSI that developed in 25 of 720 patients (3.5%) who underwent surgery for thoracolumbar degenerative spinal disorders (disc disease, spinal stenosis, and scoliosis) and were treated according to the DAIR protocol. **Results**: Of these 25 patients, 18 developed early infection (<1 month), 3 developed delayed infection (1–3 months), and 4 developed late-onset deep infection (>3 months). *Staphylococcus aureus* was isolated in 56% of the patients. The DAIR protocol was successful in 22 (88%) of the patients, while it failed in 3 (12%). Surgical implants were removed in 25% of patients with late-onset SSI, and only 11.1% with early onset and 0% with delayed SSI. All patients who failed DAIR were smokers. A significant association was found between the Charlson Comorbidity Index and the number of surgical interventions (*p* = 0.022). **Conclusions**: In this small retrospective cohort, the DAIR protocol appeared to be a feasible treatment option for deep SSI, particularly in early infections. Implant removal may be considered when infection persists after repeat DAIR or when implant loosening is observed.

## 1. Introduction

Even though basic preventive measures have been discussed thoroughly in postoperative surgical site infections (SSIs), especially in instrumented cases, treatment of deep SSI is still one of the most controversial issues in spinal surgery. SSI after instrumented spinal surgery is rare; however, potential reoperations, longer and repeated hospital admissions, extended periods of antibiotic usage, higher treatment costs, and significant morbidity and mortality continue to represent a major challenge for spine surgeons [1,2,3,4,5,6,7].

Generally, SSI is observed in 1–17% of cases but may reach up to 20% in complex instrumented spine procedures, and it has been reported as the third most frequent complication following spinal surgery, emphasizing its clinical relevance despite advances in perioperative prevention [8,9,10,11,12,13,14]. In the United States, the economic burden attributed to SSI has been estimated to range between 1 and 10 billion USD in direct and indirect medical expenditures, with approximately 8000 associated deaths annually [11]. Treatment often requires repeated hospital admissions, surgical debridement and/or implant removal, and prolonged antibiotic therapy. Late-onset SSI has been reported to be rare in the literature, with rates of 1.7–2.7% [9]. Despite meticulous contamination-control protocols and prophylactic antibiotic use, the risk of infection in the instrumented area remains clinically relevant [3].

There is no standard guideline for SSI treatment after instrumented spinal surgery because of the lack of evidence-based data. Treatment options generally consist of surgical debridement and irrigation followed by antimicrobial treatment. The irrigation and debridement procedure typically involves thorough lavage of the surgical site with antibiotic- or antiseptic-containing saline solution, combined with meticulous layer-by-layer removal of nonviable tissue, which decreases the bacterial inoculation and allows isolation of the microorganism [15]. Many studies suggest complete removal of the instruments following early- or late-onset SSI [2,10,16,17]. Retention of the implant and the decrease in antibiotic effectivity due to the biofilms over the metallic implant suggest removal of the instruments; however, doing so before achieving bone fusion to control infection can cause instability, which can result in severe back pain, radicular pain, or even neurological deficits [7,18].

The debridement, antibiotics, and implant retention (DAIR) protocol was initially described for joint infection treatment after prosthesis implantation, but was also used for deep SSI in spinal instrumentation [6,19,20]. Promising results have been reported regarding the success of the DAIR protocol, especially in early-onset (<1 month) and delayed (1–3 months) infections, which have the highest risk for non-fusion complications [4,6,16]. Removal of the instruments is usually suggested in late-onset SSI to remove the pathogens buried in the biofilm [6].

Although DAIR is increasingly used for deep SSI after spinal instrumentation, its outcomes remain insufficiently defined, particularly with respect to infection timing and implant retention. Existing studies differ substantially in infection timing, implant retention/removal strategies, and treatment protocols [6,7,16,18]. In addition, high-level evidence remains limited, making it difficult to establish standardized recommendations for specific clinical settings [8]. The present study adds a focused single-protocol experience in patients with deep SSI after thoracolumbar instrumented surgery for degenerative spinal disease, describing DAIR success, implant removal, microbiological findings, and outcomes according to infection timing.

## 2. Materials and Methods

### 2.1. Study Design, Setting, and Data Sources

This observational retrospective cohort study was conducted using clinical, surgical, microbiological, radiological, and follow-up data from patients treated at Adana City Training and Research Hospital between January 2015 and March 2025. Eligible cases were identified from institutional surgical records and the Infectious Diseases Department database. All procedures were performed at the same institution by the same senior surgeon.

### 2.2. Participants and Eligibility Criteria

We included patients aged 18 years or older with degenerative spinal disease who developed a deep SSI following instrumented thoracolumbar spinal fusion. Exclusion criteria included non-instrumented surgery, thoracolumbar stabilization performed for trauma or malignancy, and infections treated using protocols other than DAIR. Patients with a follow-up duration of less than 6 months were included in the descriptive analysis of SSI but were excluded from exploratory comparisons related to treatment failure or recurrence.

### 2.3. Definition of Deep SSI and Outcome Measures

Deep SSI was defined by a combination of the following criteria previously described in the literature [6,7]:

(I) Clinical criteria: Fever, back pain, local inflammation, and wound discharge.
(II) Laboratory findings: Elevated white blood cell count (WBC), erythrocyte sedimentation rate (ESR), C-reactive protein (CRP), and procalcitonin (PCT) levels, together with positive bacteriological cultures and/or histopathological findings.
(III) Radiological criteria (magnetic resonance imaging [MRI] and computed tomography [CT]): Evidence of infection involving deep soft tissues, muscle, and fascia, as well as findings consistent with abscess formation and spondylodiscitis.

Treatment failure (recurrence) was defined as the reappearance of signs of infection after completion of antibiotic therapy; a new SSI was defined as one caused by a pathogen different from the initial causative organism. Treatment success was defined as the absence of recurrence at 1 year, with no need for surgical revision, no clinical signs of infection, CRP < 10 mg/L, and no radiological evidence of infection. However, follow-up for a total of 2 years was suggested [6]. The Charlson Comorbidity Index (CCI) was used to evaluate the scores of patient comorbidities [21].

### 2.4. DAIR Protocol

Patients underwent skin preparation with povidone–iodine antiseptic solution (Betadine; Mundipharma, Cambridge, UK) during sterile surgical draping, and antibiotic prophylaxis with 2000 mg of cefuroxime sodium (Zinacef; GlaxoSmithKline, Brentford, UK) was administered in all procedures apart from patients with known penicillin allergies. In prolonged surgeries, the antibiotic dose was repeated every 3 h. In all cases, DAIR was performed as a deep subfascial debridement. After reopening the previous posterior incision, debridement was extended from the skin and subcutaneous tissue through the fascia into the paraspinal musculature and peri-implant space, until the pedicle screw–rod construct and bone graft bed areas were adequately visualized. Abscess cavities, necrotic tissue, infected granulation tissue, devitalized bone graft material, and biofilm-like material adherent to the implant surfaces were removed as completely as possible until macroscopically viable bleeding tissue was encountered. Tissue cultures were obtained from each layer and sent for histopathological and microbiological inspection. Infectious material was aspirated after culture sampling. Bacterial cultures and antibiotic susceptibility testing of necrotic tissue and biofilm samples were performed to guide appropriate postoperative antibiotic therapy.

Implants were retained if no loosening was detected, whereas any previously placed allografts were removed. The wound was then lavaged sequentially with 2000 mL of 0.9% sodium chloride solution (Baxter Healthcare, Medina, NY, USA), followed by rifampicin-containing saline prepared by diluting 1200 mg of rifampicin (Rifadin; Sanofi-Aventis, Paris, France) in 1000 mL of 0.9% sodium chloride solution, and finally, diluted povidone–iodine solution was prepared by diluting 35 mL of povidone–iodine (Betadine; Mundipharma, Cambridge, UK) in 1000 mL of 0.9% sodium chloride solution. A drain (Hemovac; Zimmer Biomet, Warsaw, IN, USA) was placed away from the incision site. Following these procedures, no sutures were placed in the muscle tissue. The fascia was tightly closed with interrupted polyglactin 910 absorbable sutures (Vicryl; Ethicon, Raritan, NJ, USA), and the subcutaneous tissue and skin were routinely closed with polypropylene nonabsorbable sutures (Prolene; Ethicon, NJ, USA). The drain was maintained for 5 to 10 days or until the output decreased to 20 mL per day, after which it was removed.

After initial empirical antibiotic therapy, once culture results were available, the selection and duration of systemic antibiotic treatment were determined on an individualized basis in consultation with the infectious diseases team, according to the antibiotic resistance profile of the causative organism. Surgical treatment was generally combined with appropriate pathogen-directed systemic antibiotic therapy at adequate doses for approximately 3 months. This regimen included at least four weeks of intravenous administration during hospitalization, followed by continued oral therapy after discharge.

### 2.5. Follow-Up

After discharge, patients were followed jointly with the infectious diseases team at two-week intervals. After completion of antibiotic therapy, follow-up visits were scheduled at 3–6-month intervals. Follow-up duration was recorded for each patient, and patients with insufficient follow-up were excluded from exploratory analyses of treatment failure or recurrence.

### 2.6. Bias, Study Size, and Missing Data

Because of the retrospective design, no formal calculation of sample size was performed. All eligible patients meeting the inclusion criteria during the study period were included. To reduce treatment heterogeneity, only patients treated according to the DAIR protocol were analyzed. Patients with follow-up shorter than 6 months were retained for descriptive analyses but excluded from exploratory comparisons of recurrence or treatment failure.

### 2.7. Statistical Analysis

All statistical analyses were conducted using SPSS version 23.0 (IBM Corp., Armonk, NY, USA). The normality of continuous variables was evaluated using both analytical (Shapiro–Wilk test) and visual methods (histogram inspection). Continuous variables are summarized as mean ± standard deviation (SD), median, interquartile range (IQR), and minimum–maximum values, whereas categorical variables are summarized as frequencies (*n*) and percentages (%). Comparisons between the two independent groups (single vs. multiple DAIR) were performed using the Mann–Whitney U test for continuous variables. Because of the small size of the multiple-DAIR group and low expected cell counts, categorical variables were analyzed using exact methods: nominal categorical variables were compared using Fisher’s exact test, whereas ordinal categorical variables were evaluated using the exact linear-by-linear association test for trend. A two-sided *p*-value < 0.05 was considered statistically significant. Given the small number of treatment failures, all comparative analyses were interpreted as exploratory.

### 2.8. Ethics

This study was approved by the institutional ethics committee of Adana Training and Research Hospital (approval number and date: 923—18.12.25). All patient data were anonymized before analysis.

## 3. Results

**Figure 1:**
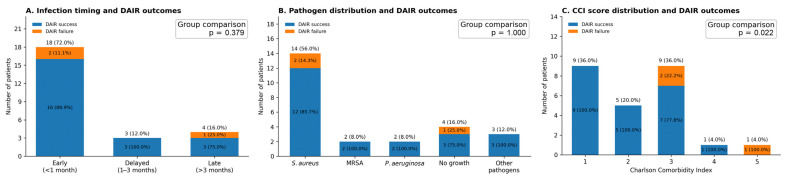
Graphical summary of infection timing, pathogen distribution, Charlson Comorbidity Index (CCI), and debridement, antibiotics, and implant retention (DAIR) outcomes in patients with deep surgical site infection (SSI). (**A**) Distribution of SSI timing with integrated DAIR outcomes, categorized as early (<1 month), delayed (1–3 months), and late (>3 months). (**B**) Distribution of pathogens with integrated DAIR outcomes; treatment failure occurred in one patient with no microbial growth and in two patients with *Staphylococcus aureus* infection. (**C**) Distribution of CCI scores with integrated DAIR outcomes. In each panel, stacked bars show DAIR success and failure, and the total number of patients with the corresponding percentage of the overall cohort is shown above each bar. Group comparison *p*-values are displayed within each panel. Group comparisons were performed using Fisher’s exact test for nominal categorical variables and the exact linear-by-linear association test for the ordinal CCI variable. CCI: Charlson Comorbidity Index, DAIR: debridement, antibiotics, and implant retention; *S. aureus*: *Staphylococcus aureus*; *P. aeruginosa*: *Pseudomonas aeruginosa*; MRSA: methicillin-resistant *Staphylococcus aureus*.

Between January 2015 and March 2025, 720 patients with thoracolumbar degenerative pathology (disc disease, spinal stenosis, and scoliosis) underwent instrumented spinal surgery. A total of 58 patients with SSI were identified in the Infectious Diseases Department database. Of these, 25 patients (3.5 %) who would be considered as deep SSI were included in the present study, and among these, 18 developed early SSI (<1 month), 3 developed delayed SSI (1–3 months), and 4 developed late-onset SSI (>3 months) (Figure 1A).

Of all patients, 15 (60%) were female and 10 (40%) were male, and the mean age was 64.12 years (range, 45–80 years). Nearly half of the patients (*n* = 12, 48%) were overweight (BMI > 25), 32% were obese (*n* = 8, BMI > 30), and only 20% (*n* = 5) had a normal body weight. Diabetes mellitus was present in 32% of patients. Seven patients (28%) had cardiovascular comorbidities (hypertension and/or heart failure), 5 (20%) had chronic pulmonary disease, and 48% (*n* = 12) were active smokers. The mean CCI was low, at 2.2. The demographic characteristics of the patient cohort are summarized in Table 1.

All patients underwent contrast-enhanced MRI and CT to assess the instrumentation. Laboratory evaluation revealed elevated WBC, ESR, CRP, and PCT levels. The mean values were as follows: CRP, 159.2 mg/L; ESR, 83.2 mm/h; PCT, 1.14 ng/mL; and WBC, 12.72 × 10^9^/L. Clinically, septic shock developed in only one of the 25 patients (4%), fever was present in 12 (48%), back pain was reported in 20 (80%), and neurological deficits were observed in 2 (8%). All patients exhibited local inflammatory wound changes and/or wound discharge.

All patients had undergone surgery for degenerative spinal disease, and seven of the 25 patients (28%) had a history of prior spinal surgery. A posterior approach was used in all cases. The mean number of instrumented spinal levels was 7.44 (range, 2–14). Interbody cages were used in 8 patients (32%), autologous bone grafts were used in 16 (64%), and allograft bone was used in 9 (36%).

Prophylactic antibiotics were administered to 23 patients (92%) with cefuroxime approximately 15 min before skin incision, while 2 (8%) received vancomycin hydrochloride (Vancocin; Eli Lilly and Company, Indianapolis, IN, USA) due to a documented penicillin allergy. The mean operative time was 171.0 min (range, 110–270 min) and the mean estimated blood loss was 434.8 mL (range, 250–700 mL). The distribution of variables is presented in Table 2.

The most common pathogen causing deep SSI was *Staphylococcus aureus*, identified in 14 patients (56%), while no microbial growth was detected in 4 (16%). The second most frequently isolated pathogens were methicillin-resistant *S. aureus* (8%) and *Pseudomonas aeruginosa* (8%) (Figure 1B). All tissue samples obtained intraoperatively were also sent for histopathological examination, which were consistent with infection.

All patients were treated with the DAIR protocol at the onset of SSI, which achieved a success rate of 88%, while treatment failure occurred in 3 patients (12%). Of these latter patients, one experienced late recurrence (1 year after the initial debridement) associated with implant loosening, and the implants were removed. In the remaining two patients, recurrent infection developed within the first month despite two applications of the DAIR protocol, necessitating implant removal due to persistent infection (Figure 1A).

Among the patients in whom DAIR failed, one had normal body weight, and two were overweight; two had a history of prior spinal surgery, and all were active smokers. With regard to surgical characteristics, the use of interbody materials was evenly distributed: one patient received an autologous graft, one an allograft, and one a combination of an allograft and cage. In terms of infection timing, two cases were early-onset, and one was late-onset SSI.

When patients who responded successfully to DAIR were compared with those who did not, smoking was more frequent in the group requiring multiple surgical interventions (100% vs. 40.9%), although this difference did not reach statistical significance (*p* = 0.096). A significant association was observed between the CCI and the number of surgical interventions (*p* = 0.022). Patients requiring multiple surgeries had higher CCI scores, and repeat surgical intervention was more frequent among those with CCI scores of 3 or higher (Figure 1C). Comparisons of variables are presented in Table 3 and Table 4.

After discharge, patients were followed jointly with the infectious diseases team at two-week intervals. After completion of antibiotic therapy, follow-up visits were scheduled at 3–6-month intervals. The mean follow-up duration was 20.56 months (range, 6–30 months), and five patients had follow-up durations of 6–12 months.

## 4. Discussion

SSI after spinal surgery is a rare complication, but one with possible mortal consequences. Greater age, obesity, diabetes mellitus, smoking, previous surgeries, extended surgical time, posterior approach, use of implants, and poor general condition of the patient and the operating room have been reported as risks of infection [12,13,14]. SSI after spinal surgery with instrumentation is a challenging issue due to the possibility of infected implants. It is vital to know how to handle deep SSI, especially because there is no certainty as to whether to remove or leave the implants in place. Traditionally, removal of spinal instrumentation has been recommended because implants may serve as a substrate for bacterial growth [2,10,16,17,22]. On the other hand, removal of the implants to control the infection prior to proper bone fusion may result in instability, back pain, radicular pain, or neurological deficits [23]. Infection treatment and retention of the implants are contradictory goals; however, preservation of the implants with the DAIR protocol has increased in popularity among recent research [1,7,19,24,25,26].

The microbiological profile of deep SSI is clinically important because the causative organism influences antimicrobial selection, biofilm-directed treatment, and the feasibility of implant retention. In the present cohort, *S. aureus* was the most frequently isolated pathogen, consistent with the meta-analysis by Zhou et al., in which *S. aureus* accounted for 37.9% of positive microbiological cultures [11]. Recent genomic evidence suggests that most SSIs after major spine surgery arise from patients’ own skin flora, often including organisms resistant to prophylactic antibiotics [27]. Similarly, Card et al. highlighted the relevance of peri-incisional skin flora in adults undergoing spine surgery by demonstrating that preoperative chlorhexidine preparation reduced skin bacterial burden during the early postoperative period [28]. These findings support the importance of obtaining multiple deep tissue and peri-implant cultures during DAIR, followed by susceptibility-guided antimicrobial therapy when implants are retained.

Varying degrees of implant retention rates have been reported in the literature. Maruo et al. reported 197 SSIs, of which 154 (78%) were treated within 90 days, and implant retention was achieved in 76% of these cases [29]. In their article, Yin et al. demonstrated that with aggressive and meticulous debridement, which includes the complete removal of biofilms stuck on the metallic surface, implant retention in 41 of 42 patients with late-onset SSI could be achieved [7]. Other authors have advocated for complete implant removal; for example, Di Silvestre et al. reported this approach in a series including 15 SSIs among 540 treated patients [25]. Similarly, Kanayama et al. reported removal of implants in 8 out of 8 patients, even in early-onset SSI [30].

The main finding of this study was a success rate of 88% with the DAIR protocol in 25 patients, with treatment failure occurring only in 12%. These results are consistent with previous studies reporting the use of this protocol for SSI treatment following spinal instrumentation. In a retrospective cohort study where 81 patients with deep SSI were treated with this protocol, implant retention was achieved in 28 of 30 patients with early-onset SSI and 19 of 51 with late-onset SSI. The cumulative 2-year survival for these patients was 71% and 84%, respectively, and the higher cumulative survival in late-onset patients when the implants were removed suggests that removing implants in these patients may be more beneficial [16]. In another article, it has been reported that 88% of the 50 patients with SSI were cured within the 2-year follow-up period [31]. In addition, the study with the highest number of patients demonstrated that DAIR has a success rate of 82% [26]. Yin et al. reported the highest rate of success in late-onset SSI with 97.6% implant retention [7]. Manet et al. treated 37 deep SSI patients with DAIR and reported a 76% success rate [6]. They suggested that DAIR is more effective in early-onset and delayed SSI with an implant retention higher than 80%, but might not be feasible in late-onset SSI patients. Hersh et al. reported in their article that instrumentation was removed in 17 of 160 patients (11%) with early infections, whereas in chronic infections, instrumentation removal was required in 99 of 172 patients (58%) [18]. General suggestions in the literature include retention of the implants in early-onset cases to preserve spinal stability, as well as removal of the implants in late-onset SSI to remove the microorganisms colonized on biofilm.

To date, there are no guidelines issued by major spine societies, nor are there level I or II evidence studies, to guide the surgical management of instrumented spinal infections. A study conducted by Lall et al. could not find level I or II evidence on the surgical treatment of spinal SSI. Retention of the implant in early-onset SSI and removal of the implant in late-onset SSI were deemed as level III evidence [8]. Even though there is no guideline, there are also no reports that show that retention of the implant hinders the treatment of early-onset SSI [32]. In our series, implant removal occurred in 11.1% of early-onset SSI and 25% of late-onset SSI; however, these estimates are based on very small subgroup numbers and should be interpreted cautiously.

Higher CCI scores were observed among patients requiring multiple surgical interventions in the present cohort. Recent reports also demonstrated that impaired host status, reflected by cumulative comorbidities, compromises immune response, wound healing, and the ability to eradicate biofilm-associated infections [33,34]. Although the association in our study reached statistical significance, only three patients experienced treatment failure; therefore, this finding should be interpreted with caution and regarded as exploratory and hypothesis-generating rather than evidence of an independent risk factor. Nevertheless, this observation suggests that systemic comorbidity burden may influence DAIR outcomes and should be evaluated in larger multicenter studies with adequate event numbers.

The present study has several limitations, most importantly the small sample size and the very low number of treatment failures requiring more than one surgical intervention (*n* = 3), resulting in a markedly imbalanced group distribution (22 vs. 3) and limited statistical power for both continuous and categorical comparisons. Because only three failures occurred, logistic regression (including multivariable modelling) was not methodologically appropriate, as such models would be unstable, prone to overfitting, and yield imprecise estimates with wide confidence intervals. Similarly, the low event count precluded meaningful survival analyses (e.g., Kaplan–Meier curves), which would lack discrimination and produce unreliable estimates. Therefore, the findings should be interpreted as primarily descriptive and hypothesis-generating rather than definitive.

Future studies should aim to validate these findings in larger, preferably multicenter cohorts with standardized definitions of infection timing, DAIR success, recurrence, and implant removal. Prospective data collection would allow more reliable evaluation of patient-related, microbiological, radiological, and surgical predictors of DAIR failure, including comorbidity burden, smoking status, pathogen type, antibiotic regimens, implant loosening, and fusion status. In addition, comparative studies evaluating DAIR, staged implant exchange, and implant removal according to infection timing may help define more individualized treatment algorithms for deep SSI after spinal instrumentation.

## 5. Conclusions

The management of late-onset deep SSI following instrumented spinal surgery remains challenging, and current treatment strategies vary considerably, including implant retention, exchange, or removal. In the present retrospective cohort, the DAIR protocol appeared to be a feasible treatment strategy, particularly for early-onset and delayed SSI. However, because treatment failure occurred in only three patients, the observed associations between smoking status, CCI, and DAIR failure should be interpreted with caution and regarded as exploratory and hypothesis-generating rather than definitive risk factors. Further multicenter studies with larger cohorts and adequate event numbers are required to validate these observations and to support the development of standardized treatment recommendations.

## Figures and Tables

**Table 1:** The demographic characteristics of the patient cohort. IQR: interquartile range, BMI: body mass index, ESR: erythrocyte sedimentation rate, CRP: C-reactive protein, WBC: white blood cells, PCT: procalcitonin.

Variable	Mean ± SD	Median	IQR	Min–Max
Age (Year)	64.12 ± 8.23	65	12	45–80
BMI (kg/m^2^)	28.72 ± 4.08	27.5	7	23–37
Operation time (min)	171.0 ± 45.16	160	35	110–270
Blood loss (mL)	434.8 ± 115.3	410	150	250–700
ESR (mm/h)	83.2 ± 21.55	80	35	40–120
CRP (mg/L)	159.2 ± 47.43	160	65	80–270
WBC (K/µL)	12.72 ± 1.33	13	2	9–15
PCT (ng/mL)	1.14 ± 0.80	1.10	0.75	0.40–4.50
Duration of antibiotic treatment (weeks)	13.84 ± 3.48	14	6	8–20
Follow-up (months)	20.56 ± 6.77	24	10	6–30

**Table 2:** The distribution of variables among all patients. BMI: body mass index, SSI: surgical site infection.

Variable	Category	*n*	%
Sex	Male	10	40.0
	Female	15	60.0
BMI Category	Normal	5	20.0
	Overweight	12	48.0
	Obese	8	32.0
Interbody Cage/Graft	Autologous Graft	14	56.0
	Allograft	3	12.0
	Allograft + Interbody Cage	6	24.0
	Autologous Graft + Interbody Cage	2	8.0
SSI Timing	Early	18	72.0
	Delayed	3	12.0
	Late	4	16.0
Pathogen	No pathogen	4	16.0
	*Staphylococcus aureus*	14	56.0
	Methicillin-resistant *Staphylococcus aureus*	2	8.0
	*Pseudomonas aeruginosa*	2	8.0
	*Klebsiella*	1	4.0
	*Escherichia coli*	1	4.0
	*Proteus*	1	4.0
Charlson Comorbidity Index Scores	1	9	36.0
	2	5	20.0
	3	9	36.0
	4	1	4.0
	5	1	4.0

**Table 3:** Comparisons of demographic characteristics between single and multiple surgery patients. IQR: interquartile range, BMI: body mass index, ESR: erythrocyte sedimentation rate, CRP: C-reactive protein, WBC: white blood cells, PCT: procalcitonin.

	Single Surgery (*n* = 22)			Multiple Surgeries (*n* = 3)			
Variable	Mean ± SD	Median (IQR)	Min–Max	Mean ± SD	Median (IQR)	Min–Max	*p*-Value
Age (Year)	64.00 ± 8.42	65 (12)	45–80	65.00 ± 8.19	67 (—)	56–72	0.844
BMI (kg/m^2^)	29.05 ± 4.20	27.75 (7)	23–37	26.33 ± 2.08	27 (—)	24–28	0.446
Operation time (min)	172.5 ± 47.98	160 (54)	110–270	160.0 ± 10.0	160 (—)	150–170	0.969
Blood loss (mL)	439.1 ± 122.24	425 (165)	250–700	403.3 ± 32.15	390 (—)	380–440	0.783
ESR (mm/h)	83.64 ± 22.16	85 (33)	40–120	80.0 ± 20.0	80 (—)	60–100	0.783
CRP (mg/L)	157.27 ± 50.26	150 (75)	80–270	173.33 ± 11.55	180 (—)	160–180	0.353
WBC (K/µL)	12.68 ± 1.38	13 (2)	9–15	13.0 ± 1.00	13 (—)	12–14	0.783
PCT (ng/mL)	1.17 ± 0.85	1.05 (0.75)	0.40–4.50	0.93 ± 0.38	1.10 (—)	0.50–1.20	0.783
Duration of antibiotic treatment (weeks)	13.73 ± 3.64	13 (6)	8–20	14.67 ± 2.31	16 (—)	12–16	0.497
Follow-up (months)	20.73 ± 6.25	22 (10)	8–30	19.33 ± 11.72	24 (—)	6–28	0.969

**Table 4:** Comparisons of variables between single and multiple surgery patients. BMI: body mass index, SSI: surgical site infection.

Variable	Category	Single Surgery (*n* = 22)	Multiple Surgeries (*n* = 3)	*p*-Value
Sex	Male	9 (40.9)	1 (33.3)	1.000
	Female	13 (59.1)	2 (66.7)	
BMI Category	Normal	4 (18.2)	1 (33.3)	0.416
	Overweight	10 (45.5)	2 (66.7)	
	Obese	8 (36.4)	0 (0)	
Interbody Cage/Graft	Autologous Graft	13 (59.1)	1 (33.3)	0.486
	Allograft	2 (9.1)	1 (33.3)	
	Allograft + Interbody Cage	5 (22.7)	1 (33.3)	
	Autologous Graft + Interbody Cage	2 (9.1)	0 (0)	
SSI Timing	Early	16 (72.8)	2 (66.7)	0.379
	Delayed	3 (13.6)	0 (0)	
	Late	3 (13.6)	1 (33.3)	
Pathogen	No Pathogen	3 (13.6)	1 (33.3)	1.000
	*Staphylococcus aureus*	12 (54.5)	2 (66.7)	
	Methicillin-Resistant *Staphylococcus aureus*	2 (9.1)	0 (0)	
	*Pseudomonas aeruginosa*	2 (9.1)	0 (0)	
	*Klebsiella*	1 (4.5)	0 (0)	
	*Escherichia coli*	1 (4.5)	0 (0)	
	*Proteus*	1 (4.5)	0 (0)	
Charlson Comorbidity Index Scores	1	9 (40.9)	0 (0)	0.022
	2	5 (22.7)	0 (0)	
	3	7 (31.8)	2 (66.7)	
	4	1 (4.5)	0 (0)	
	5	0 (0)	1 (33.3)	

## Data Availability

The raw data supporting the conclusions of this article will be made available by the authors on request.

## Abbreviations

The following abbreviations are used in this manuscript:

Abbreviation	Full form
BMI	Body mass index
CCI	Charlson comorbidity index
CRP	C-reactive protein
CT	Computed tomography
DAIR	Debridement, antibiotics, and implant retention
ESR	Erythrocyte sedimentation rate
IQR	Interquartile range
MRI	Magnetic resonance imaging
PCT	Procalcitonin
SD	Standard deviation
SSI	Surgical site infection
WBC	White blood cell count
